# Wilms tumor in adults—an unusual encounter: a case report

**DOI:** 10.1186/s13256-025-05776-0

**Published:** 2026-02-02

**Authors:** Neeraj Kolap, Ratnadip Sonawane, Mayur Baviskar, Mayur Bhalghat

**Affiliations:** PCMC PGI YCM Hospital, Pune, Pimpri, 411018 India

**Keywords:** Adult Wilms tumor, Nephrectomy, Nephroblastoma, Renal malignancy, Adult onset nephroblastoma

## Abstract

**Background:**

Wilms tumor, or nephroblastoma, arises from embryonal metanephric blastemal cells and is the most common renal malignancy in the pediatric population. However, its occurrence in adults is extremely rare, with only a limited number of cases documented in the literature. In adults, renal masses most commonly turn out to be renal cell carcinomas, which can closely mimic Wilms tumor on imaging, posing a significant diagnostic challenge. Particularly in resource-limited settings, definitive diagnosis is often established only after histopathological evaluation of nephrectomy specimens.

**Case presentation:**

We present the case of a 24-year-old Indian woman who presented with a progressively enlarging lump in the right flank. The subsequent investigations revealed a right renal mass, suspected to be malignant, for which the patient underwent a right radical nephrectomy. Histopathological examination confirmed the diagnosis of adult Wilms tumor (nephroblastoma).

**Conclusion:**

This case highlights the rare occurrence of Wilms tumor in an adult female and underscores the diagnostic challenges it poses. We aim to contribute to the limited body of literature on adult nephroblastoma and promote awareness of this rare entity among clinicians and pathologists.

## Introduction

Wilms tumor, or nephroblastoma, is named after Carl Max Wilhelm Wilms, a nineteenth century German surgeon. It arises from the primitive metanephric blastema of the kidneys and is the most common malignant tumor originating from kidneys in the pediatric population [1]. It is most commonly seen in children under the age of 5 years, with the highest incidence occurring between children aged 3 and 4 years. Studies indicate that the median age of onset for tumors in children is 38 months, with no significant reports highlighting gender differences [2]. Wilms tumor is rarely seen in adults (more than 16 years of age) accounting for less than 1% of all kidney cancers, with an incidence of 0.2 cases per million people per year [2, 3].

Adults may typically present with abdominal pain and hematuria, whereas in children, the tumor often appears as a painless, rapidly growing abdominal mass that is easily palpable [1]. The lungs and liver are common sites of distant metastasis; however, metastasis to the bone, skin, bladder, large intestine, central nervous system, and contralateral kidney is uncommon [1, 4]. While childhood Wilms tumors are often identified through ultrasound, their radiological appearance can be similar to that of more common adult malignant renal neoplasms, such as renal cell carcinoma (RCC) [3]. Abdominal ultrasonography (USG) is an effective screening tool for determining renal or extrarenal origin of mass. A contrast enhanced computed tomography (CECT) scan of the abdomen or magnetic resonance imaging (MRI) can provide further information about the position of the tumor [4].

Histologically, the defining features of “favorable” Wilms tumors include the presence of blastemal, epithelial, and mesenchymal cells. Wilms tumors characterized with “unfavorable” histology show a significant level of anaplasia [2]. Wilms tumors are referred to as “triphasic,” as they consist of blastemal, epithelial, and stromal elements. However, it is common for only one or two components to predominate. There are no histological differences between Wilms tumors in children and those in adults [3]. According to study by Kilton *et al*., for a tumor to be called an adult Wilms tumor the following criteria should be met: (a) the tumor has to be a primary renal neoplasm, (b) the presence of round cells or primitive blastemic spindle components, (c) the presence of abortive or embryonal tubules or glomerular structures, (d) the absence of typical areas diagnostic of renal cell carcinoma, (e) the pictorial confirmation of histology, and (f) the patient’s age should be > 15 years [1, 5].

Adult Wilms tumors harbor genetic alterations previously reported in the more common pediatric Wilms tumors, including WT1 mutations, ASLX1 mutations, NSD2 mutations, and 11p loss. A significant subset of adult Wilms tumors (specifically those of epithelial type with differentiated areas) harbor targetable BRAF V600E mutations and appear to arise from metanephric adenomas as a consequence of additional acquired genetic alterations [6].

Treatment varies depending on factors such as age, stage, pathology (whether favorable or anaplastic), response to therapy, and genetic status [4]. Patients receive treatment according to protocols established for children by the National Wilms Tumor Study (NWTS), now known as the Children’s Oncology Group (COG), in North America and the International Society of Paediatric Oncology (SIOP) in Europe. The treatment guidelines typically include a combination of surgery, chemotherapy, and, in some cases, radiotherapy [7]. Radical nephrectomy, which involves removing the tumor along with the kidney, adrenal gland, and lymph nodes on the same side, is the preferred treatment for unilateral nephroblastoma [7]. Data indicate that neoadjuvant and/or adjuvant chemotherapy combined with surgery (with or without radiotherapy) enhances survival rates for the majority of patients with Wilms tumor [7]. This case report was reported in accordance with the CARE checklist.

## Case report

### Presentation

A 24-year-old Indian woman, a housewife from a nearby village, presented to the surgery outpatient department of our tertiary care hospital with a 4-month history of abdominal distension and discomfort. She had delivered a healthy female child 4 months prior and initially attributed her symptoms to post-pregnancy changes. However, when the symptoms persisted with no improvement after delivery, she sought medical evaluation. The patient reported a sensation of fullness on the right side of her abdomen. She denied any abdominal pain, nausea, vomiting, fever, jaundice, altered bowel habits, or hematuria. The patient had no comorbidities or addictions. She has two healthy children, both delivered full term at home without complications, assisted by a local midwife.

**Fig. 1:**
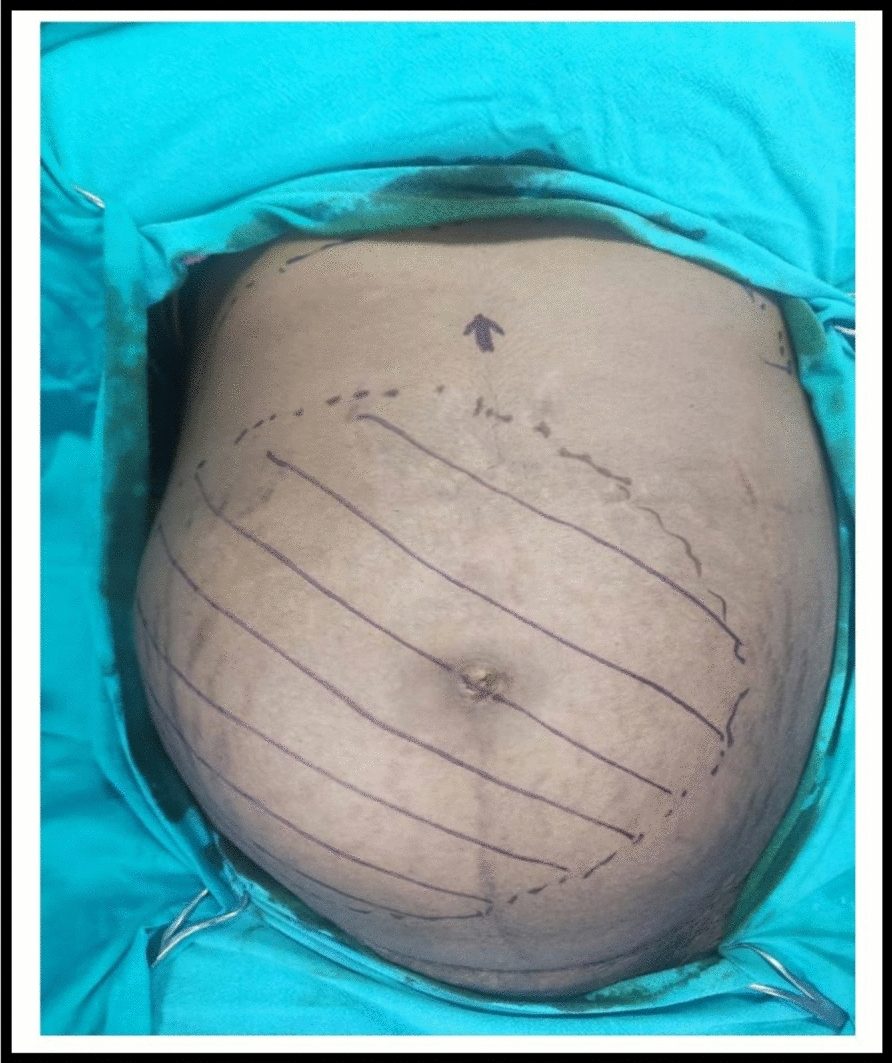
Shows palpable limit of the tumor on abdominal examination

On examination, the patient was vitally stable. Abdominal examination revealed a single, ovoid-shaped palpable lump on the right side of the abdomen, extending from the right subcostal region to the pelvis. The lump was firm to cystic in consistency, nontender on palpation, and moved with respiration. Vaginal and rectal examinations showed no significant findings. The remaining panel of relevant blood work and urine investigations were within normal limits, revealing no apparent pathology (Fig. 1).

### Investigations

Further work-up in the form of radiological investigations including abdominal USG and CECT scan revealed a 25 cm × 15 cm × 15 cm sized, large lobulated heterogeneous solid cystic retroperitoneal lesion in the right side of the abdomen extending from the inferior surface of right lobe of liver to the right iliac fossa. The right kidney and adrenal gland could not be appreciated separately. A diagnostic preoperative biopsy was deferred owing to the risk of tumor spillage into the abdominal cavity, particularly given the cystic nature of the lesion on imaging.

**Fig. 2:**
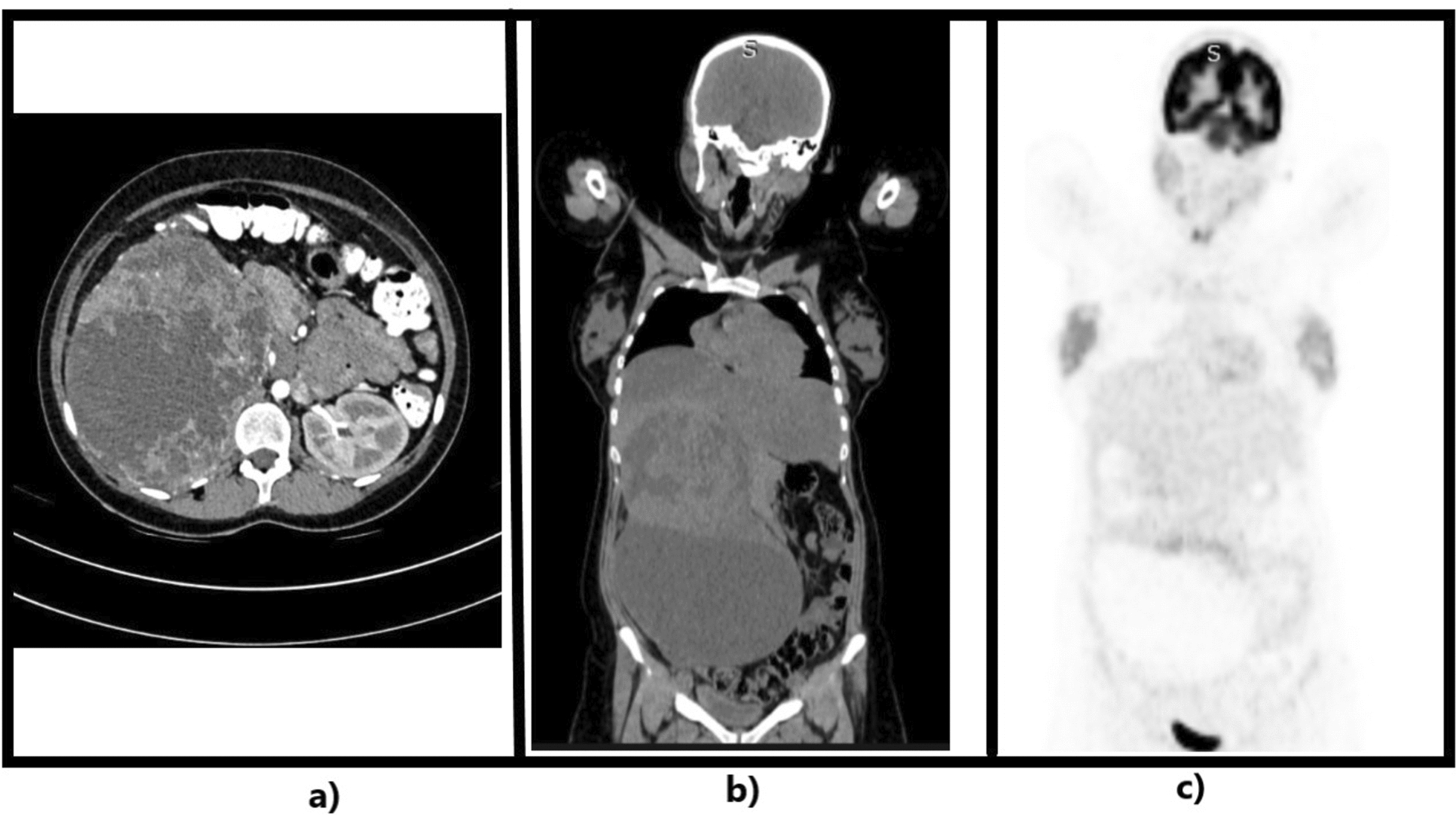
**a** Axial contrast enhanced computed tomography films showing a mass arising from the right kidney and a normal left kidney. **b** Coronal contrast enhanced computed tomography films showing the extent of the tumor. **c** Positron emission tomography and computed tomography films showing no evidence of metastasis

On primary suspicion of a malignant renal tumor, we further ordered a positron emission tomography (PET) scan to rule out any distant metastasis, the reports showed no evidence of fluorodeoxyglucose (FDG) avid distant organ involvement. The renal scans for assessment of normal functioning of contralateral kidney could not be done owing to high cost of the investigation, but the renal function tests of the patient were fairly normal with blood urea levels (BUL ) of 12 µg/dL and serum creatinine of 0.51 µg/dL (Fig. 2).

### Surgery

**Fig. 3:**
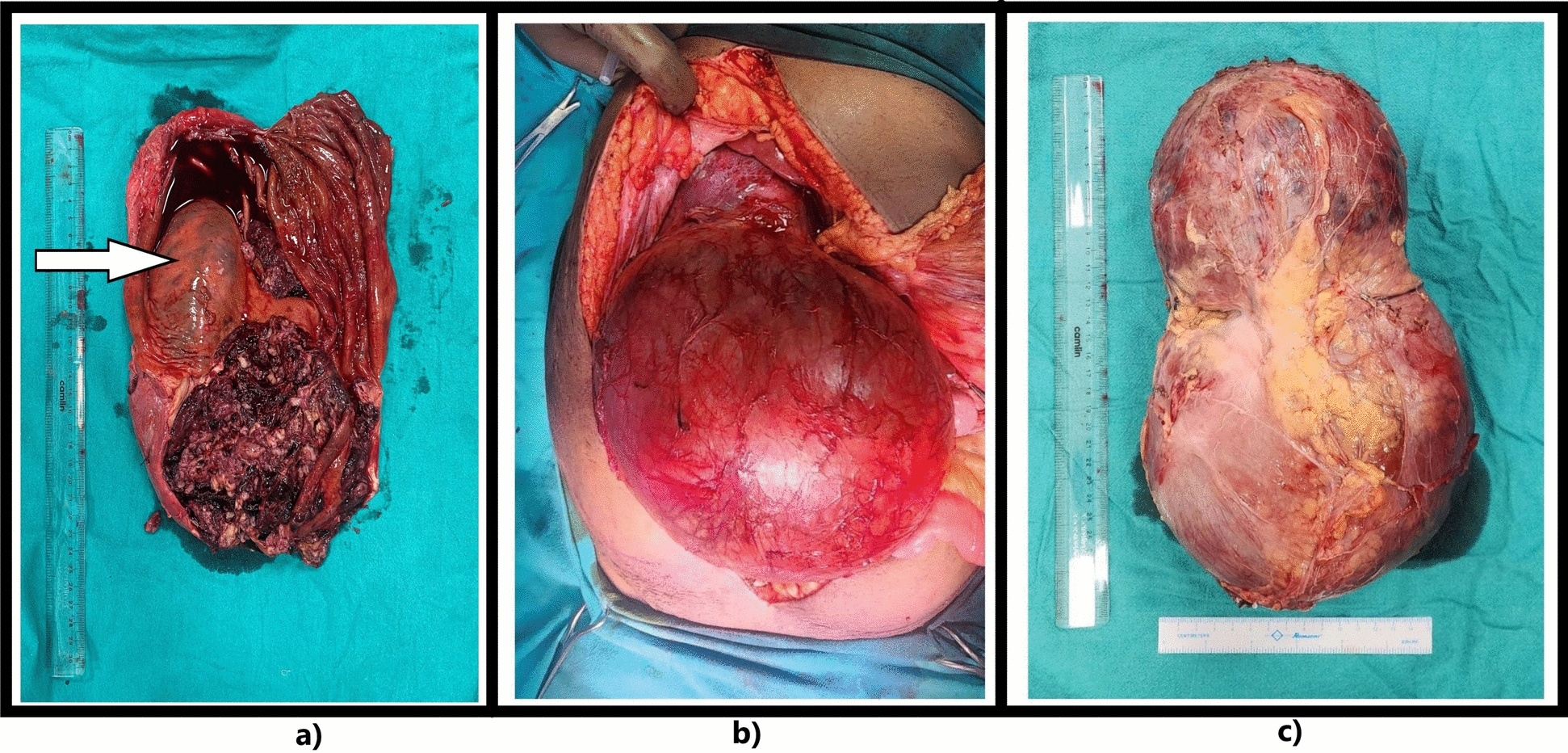
**a** Cut section of the tumor showing the right kidney marked by an arrow. **b**, **c** Postoperative excised tumor specimen

After requisite preoperative optimization, the patient was posted for elective exploratory laparotomy and intraoperatively found a tumor weighing 6 kg, sized 25 cm × 15 cm × 15 cm, arising from the right kidney, which was adherent to the inferior surface of the right lobe of the liver and inferior surface of the right hemidiaphragm at its cranial end and extending into the pelvis towards its caudal end (Fig. 3). The tumor was adherent to the inferior surface of the right hemidiaphragm, adhesiolysis was performed and the defect in diaphragm was closed primarily with prolene sutures. The tumor was seen to be compressing on the pancreas, the duodenum, and the inferior vena cava, and the renal vessels were drawing a significant vascular supply from the vessels without direct invasion of the inferior vena cava. A right radical nephrectomy was done. The patient was stable and tolerated the procedure well and was shifted to the surgical intensive care unit (ICU) for postoperative monitoring.

The tumor specimen, measuring approximately 25 cm × 15 cm × 12 cm, appeared solid–cystic with gray–white and brown areas. The cut section revealed interspersed solid and cystic regions, with the solid component (40% of the tumor) located opposite the renal sinus, sharply demarcated from the right kidney (15 cm × 12 cm), showing extensive hemorrhage and blood clots. A stretched adrenal gland, unaffected by the tumor, was located at the cranial end. The right kidney, measuring 9.5 cm × 9 cm × 5 cm, was eccentrically present within the cyst.

**Fig. 4:**
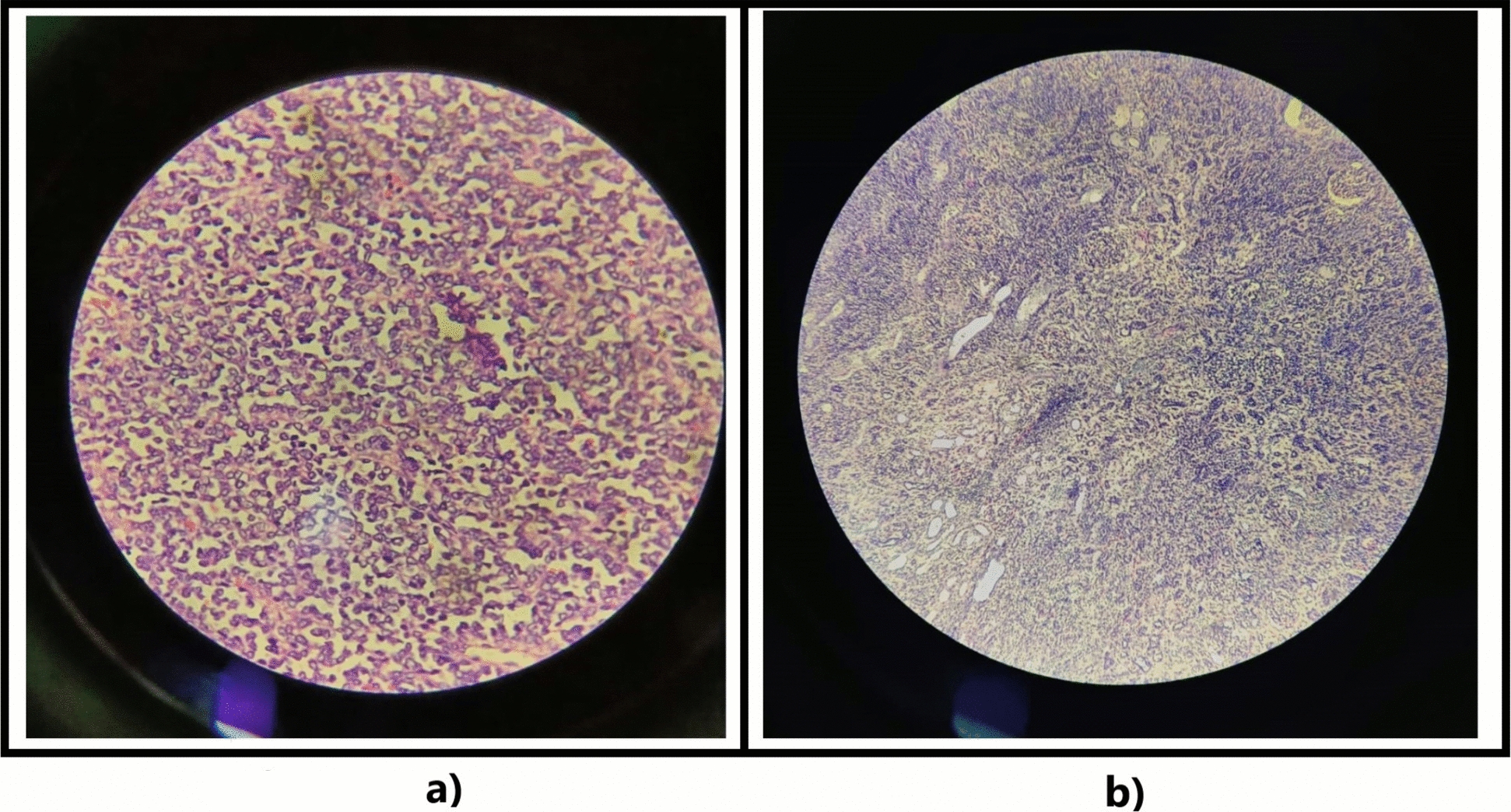
(**a**) High magnification view and (**b**) low magnification view of slides showing the biphasic tumor with a component of blastemal cells, which consists of small-to-medium sized undifferentiated cells with relatively small regular nuclei and small nucleoli arranged in a diffuse pattern. The epithelial component includes poorly differentiated cells (rosette-like structures)

The microscopic examination revealed a biphasic nephroblastoma (epithelial and blastemal) without anaplastic features. The surgical margins were clear, and lymph node specimens showed no involvement. According to the WHO 5th edition (2021), SIOP, and COG, the tumor is classified as stage I: tumor limited to the kidneys, completely resected with intact renal capsule, renal sinus, and the renal vessels, and ureter free of tumor (Fig. 4).

### Outcome

The patient recovered well over her course of stay in the hospital and was discharged with advice to seek a medical oncologist’s opinion. At present, the patient is now taking chemotherapy as advised by oncologists without any recurrence of symptoms and is in a stable condition.

## Discussion and conclusion

### Literature review

There is a limited number of documented cases of adult Wilms tumor available online owing to the rare occurrence of this tumor in adults. Wilms tumor is very rare in adults, with an incidence of about 0.2 per million per year, and represents less than 1% of all diagnosed renal tumors [1]. Hence we publish this report to document our encounter with this unlikely occurrence of Wilms tumor in adults and contribute to the limited literature available online: a Malawian report from 2019 suggests only ten such reports exist.

Our patient, a resident of a rural area in India with limited access to specialized diagnostic and treatment facilities, presented with complaints of abdominal discomfort and distension. Initially, she attributed these symptoms to changes following her recent pregnancy. However, she became concerned when her abdomen failed to return to its pre-pregnancy size even 4 months after delivery. Upon evaluation, these symptoms were found to be due to a large retroperitoneal mass arising from the right kidney. Given existing reports of Wilms tumor presenting during pregnancy [8–10], we inquired about any similar symptoms during her gestation, but the patient reported no such complaints prior to or during her pregnancy. We started optimization of the patient for radical nephrectomy with a presumptive diagnosis of renal cell carcinoma.

### Diagnosis

In adults, renal cell carcinoma is the most common differential diagnosis for retroperitoneal renal masses. In contrast, nephroblastoma (Wilms tumor) is a rare consideration and is typically confirmed only after histopathological evaluation, as in our case. While image-guided preoperative biopsy can facilitate early diagnosis of adult Wilms tumor, it is often avoided owing to the potential risk of tumor spillage into the peritoneal cavity, particularly when malignancy is suspected. Although molecular genetic studies could have provided valuable insights into targetable mutations, they were not performed owing to their high cost and limited availability at our center.

Wilms tumor is believed to arise from persistent nephrogenic rests or metanephric tissue, though its etiology remains unknown [2]. The syndromic association of Wilms tumor is uncommon in adults but is usually seen in children. WAGR syndrome is characterized by the presence of Wilms tumor, aniridia, genitourinary abnormalities, and intellectual disability. Children with WAGR syndrome have a 50% risk of developing Wilms tumor, owing to a chromosomal abnormality in the WT1 gene. Denys–Drash syndrome, also known as Drash syndrome, is another condition linked to Wilms tumor. It is characterized by male pseudohermaphroditism and early-onset renal failure, often beginning in infancy [2].

### Treatment and prognosis

Wilms tumors are usually treated with a multimodal approach with surgery, chemotherapy and/or radiotherapy [1]. The Children’s Oncology Group (COG), previously known as NWTS, and the International Society of Pediatric Oncology (SIOP) have two acceptable treatment protocols for application in adults after adjustment of the pediatric protocols [2, 4]. The NWTS has consistently recommended initial nephrectomy to determine the precise stage of the tumor and its histology, which then guides further treatment decisions. The SIOP investigators introduced the concept of pre-nephrectomy chemotherapy for all patients over 6 months of age, to shrink the tumor, reduce the risk of intraoperative rupture and spillage, and increase the likelihood of achieving a lower tumor stage, ultimately requiring less intensive treatment [1].

The findings from NWTS-5 indicate that adjuvant chemotherapy can be safely omitted for children under 2 years of age at diagnosis with a stage I favorable histology (FH) Wilms tumor, provided the tumor weighs less than 550 g [7]. The most commonly used treatment for stage I intermediate-risk, stage II and III low-risk, stage II–IV intermediate-risk, and stage I high-risk Wilms tumors involves a combination of vincristine, actinomycin D, and doxorubicin (Adriamycin). For stages II–IV with unfavorable high-risk histology, the recommended regimen includes carboplatin/etoposide and cyclophosphamide-doxorubicin (CYCLO-ADR) [7]. The indications for radiotherapy are macroscopic residual disease after surgery, recurrent abdominal disease, pulmonary metastasis, liver metastasis, skeletal metastasis, and unresected LN metastasis, not recommended in stage I/II with a favorable histology [7].

Histopathological examination revealed a stage I biphasic Wilms tumor with favorable histology and no evidence of anaplasia in our case. Following successful radical nephrectomy, the patient is currently undergoing adjuvant chemotherapy with a combination of vincristine and actinomycin D, as recommended by the oncology team. This case highlights the importance of maintaining a high index of suspicion for Wilms tumor in adult patients presenting with renal masses, and aims to raise awareness among surgeons about this rare but significant differential diagnosis. The use of modern technology can help enhance the recovery from this serious illness.

## Data Availability

Not applicable.
